# Biomarker integration for improved biodosimetry of mixed neutron + photon exposures

**DOI:** 10.1038/s41598-023-37906-3

**Published:** 2023-07-06

**Authors:** Igor Shuryak, Shanaz A. Ghandhi, Evagelia C. Laiakis, Guy Garty, Xuefeng Wu, Brian Ponnaiya, Emma Kosowski, Evan Pannkuk, Salan P. Kaur, Andrew D. Harken, Naresh Deoli, Albert J. Fornace, David J. Brenner, Sally A. Amundson

**Affiliations:** 1 Center for Radiological Research, Columbia University Irving Medical Center, 630 West 168Th Street, VC-11-234/5, New York, NY 10032 USA; 2 Department of Oncology, Lombardi Comprehensive Cancer Center, Georgetown University, Washington, DC USA; 3 Department of Biochemistry and Molecular & Cellular Biology, Georgetown University, Washington, DC USA

**Keywords:** Biophysics, Computational biology and bioinformatics, Systems biology

## Abstract

There is a persistent risk of a large-scale malicious or accidental exposure to ionizing radiation that may affect a large number of people. Exposure will consist of both a photon and neutron component, which will vary in magnitude between individuals and is likely to have profound impacts on radiation-induced diseases. To mitigate these potential disasters, there exists a need for novel biodosimetry approaches that can estimate the radiation dose absorbed by each person based on biofluid samples, and predict delayed effects. Integration of several radiation-responsive biomarker types (transcripts, metabolites, blood cell counts) by machine learning (ML) can improve biodosimetry. Here we integrated data from mice exposed to various neutron + photon mixtures, total 3 Gy dose, using multiple ML algorithms to select the strongest biomarker combinations and reconstruct radiation exposure magnitude and composition. We obtained promising results, such as receiver operating characteristic curve area of 0.904 (95% CI: 0.821, 0.969) for classifying samples exposed to ≥ 10% neutrons *vs*. < 10% neutrons, and R^2^ of 0.964 for reconstructing photon-equivalent dose (weighted by neutron relative biological effectiveness) for neutron + photon mixtures. These findings demonstrate the potential of combining various -omic biomarkers for novel biodosimetry.

## Introduction

In the current global political situation, there continues to be the potential of large-scale exposures of people to ionizing radiation. Physical radiation dosimetry devices are not present among the general population, and consequently there is an important need to develop and perfect biodosimetry assays^1–3^ to reliably estimate the radiation dose absorbed by each exposed person based on easily accessible samples of biological materials such as blood, urine and/or saliva. Moreover, biodosimetry needs to predict not only the absorbed dose, but also the severity of corresponding biological injuries (*e.g.* hematopoietic and intestinal damage).

Realistic radiation exposures from nuclear weapons and reactor accidents will involve a complex mixture of radiation types, including photons and neutrons. The neutron components vary in magnitude between individuals due to distance from the source and shielding by objects, which are penetrated to different extents by different radiations. Due to different patterns of microscopic energy deposition in irradiated tissues relative to photons, neutrons are likely to have profound impacts on radiation-induced disease type and progression, and different medical countermeasures may be required for neutron-induced disease.

Multiple biological signals, including genes, metabolites, blood cell counts, and cytogenetic damage, are being developed as radiation-responsive biomarkers. Integration of several such biomarker types into a comprehensive quantitative modeling framework can improve biodosimetry by enabling the strengths of different biomarkers to complement each other, and weaknesses to partially cancel out. For example, different biomarkers may have better performance in different dose ranges – some at low doses and others at higher doses. We showed that combining different biomarkers can provide superior dose reconstruction accuracy, compared with a single biomarker^4–6^.

Biodosimetry workflows utilizing a systems biology approach can combine multiple “-omic” biomarkers using machine learning (ML) algorithms. This approach can also readily integrate other types of data inputs in addition to biomarkers, such as demographic data on the exposed individuals or blood cell counts. In the current study, we investigated ML-based approaches to improve biodosimetry in complex exposure scenarios such as neutron + photon mixtures in various proportions, using combinations of several biomarker classes obtained from different biofluids: gene expression, urine and serum metabolites, and blood cell counts. Such a combined strategy could be particularly useful in realistic scenarios for mass-casualty events, where the population of potentially irradiated individuals is very heterogeneous in terms of exposure dose and neutron proportion. Our study supports the innovative concept of combining data from several radiation-responsive biological signals to improve biodosimetry performance.

## Materials and methods

### Experimental procedures

C57BL/6 mice from Charles River laboratory (CRL) (Frederick, MD) were irradiated with neutrons, x-rays, or a mixture of photons and neutrons up to 3 Gy total dose. Irradiations were conducted at the Columbia IND neutron Facility, with dosimetry performed daily as described elsewhere^7^. Neutrons were generated by impinging a mixed beam of 5 MeV protons, deuterons and molecular ions on a thick beryllium target. Up to 18 mice were placed in modified 50 ml centrifuge tubes on a Ferris wheel rotating around the target. 9–10 µA of beam was used to generate about 1 Gy/h of neutrons with a concomitant dose of 0.2 Gy/h of photons. To accommodate flipping the mice front-to-back half way through each dose, dose was delivered in fractions of 0.15 Gy neutrons (+ 0.034 Gy concomitant photons), with mice receiving 2, 4, 6 or 16 fractions, corresponding to 0.3, 0.6, 0.9 or 2.4 Gy of neutrons (10%, 20%, 30% or 80% of 3 Gy). Mice made approximately 5 full revolutions around the target for each fraction.

Following neutron irradiations, an X ray boost was delivered individually to each mouse, using an RS-2000 small animal irradiator (Rad Source, Buford, GA; 160 kVp, 25 mA, 1 Gy/min). The RS-2000 utilizes a 0.3 mm Cu filter resulting in a 1 mm Cu Half Value Layer. The boost dose was calculated for each set of mice to result in a total dose (Neutrons + concomitant photons + X rays) of 3 Gy. Dosimetry was performed on the day of the experiment, using a factory calibrated ionization chamber (Radcal 10 × 6–6; Monrovia, CA) calibrated to air Kerma. All animal studies were approved by the Columbia Institutional Animal Care and Use Committee (IACUC approved protocol # AABR9603) and were conducted in facilities accredited by the Association for Assessment and Accreditation of Laboratory Animal Care (AAALAC) and followed all relevant federal and state guidelines. The study is reported in accordance with ARRIVE guidelines ([https://arriveguidelines.org).

Unirradiated control mice were subjected to the same handling, including insertion in the tubes and rotation for a similar amount of time on a second ferris wheel placed outside the accelerator hall. Sham control and x-ray only (0% neutrons) exposed mice were euthanized 1 or 7 days after irradiation and whole blood collected by cardiac puncture. Blood was mixed with PAXgene solution (Becton Dickinson) and mixed by inverting 10X, then stored at -80ºC overnight. Samples were thawed at room temperature and then stabilized for > 2 h before RNA isolation using the PAXgene kit (Qiagen). RNA was quantified using the Nanodrop One (Thermofisher) and quality was assessed by the A_260/280_ ratios. One microgram of total RNA from all samples was reverse transcribed and gene expression measured and analyzed following the methods described in detail in reference^8^. Specifically, a “net signal” for genes was generated by calculating the difference between geometric means of the gene group that was upregulated after radiation exposure, and the group that was downregulated after exposure. Blood cell counts were obtained using a CytoFLEX flow cytometer (Beckman Coulter Inc., Brea, CA), as previously described^8^.

Serum and spot urine samples were collected and processed with identical conditions to previously published work from our group^9^. Small molecule profiles were obtained through Ultra Performance Liquid Chromatography time-of-flight mass spectrometry (Waters UPLC Xevo G2 QTOF), as previously described, and data were deconvoluted with Progenesis QI (NonLinear Dynamics, Newcastle UK). Positive identifications of candidate metabolites from the previously published biosignature were obtained through tandem mass spectrometry by matching of fragmentation patterns in a quality control sample to those of pure standards. Data normalization for metabolomic data was conducted as described previously^9^. Only data of previously characterized biomarkers were included in the current analysis, comprising of 12 metabolites in urine and 17 in serum. The mice were euthanized with 3 L/min of CO2 for 5 min then cervical dislocation.

### Data preparation and processing

We matched gene expression qRT-PCR data (genes) and metabolomic data sets for 89 mice, using serum and urine samples collected 1 or 7 days after radiation exposure. This data set represents a subset from the data base collected by our institutions (Columbia and Georgetown Universities): there were 239 mice with metabolomics data, 103 mice with transcriptomics data, and 89 mice with both types of data.

Since the amount of blood that could be safely drawn from each mouse without causing severe morbidity or mortality was limited, it was technically not possible to collect enough blood from any one mouse to perform both the blood cell counts analysis, and the metabolomics and transcriptomics analyses. Due to this technical limitation, blood cell counts data were also available, but not from the same animals as the transcriptomics and metabolomics data.

We addressed this limitation in two ways: (1) We combined the blood cell count data with the other data by random matching of individuals. We believe that this procedure is justified because the goal of our study was to integrate all available biomarkers, and the blood cell count data typically varied relatively little between animals in the same exposure group. For example, the coefficient of variation (CV) between mice for ln-transformed CD19 cell counts across all mouse groups had a median value of only 16.3%, with a range of 2.8 to 39.3%. The resulting combined data were analyzed as described below. (2) Alternatively, we excluded the CD19 cell counts variable from the analysis, and performed biodosimetry calculations without this variable.

For transcript data, we used the signal (C_T_ value) for each candidate gene separately, and also calculated a net signal (difference in geometric means between upregulated and downregulated radiation-responsive genes), as in our previous analyses^8^. The gene net signal in this case was composed of 7 genes. There were 3 genes for which C_T_ values decreased with dose: Lrg1, Phlda3, and Rhoc. There were 3 genes for which C_T_ values increased with dose: CCr7, Cd19, Cxcr5, and Ly6d. The gene net signal was the geometric mean of the first group of 3 genes, minus the geometric mean of the second group of 4 genes. For blood cell counts, we used ln-transformed values of each cell type (CD45, CD3e, Ly_6G, and CD19), and also calculated the ln-transformed neutrophil/lymphocyte ratio (Ly_6g/CD45). The combined data set is provided in Supplementary File 1 online (full_data_set tab).

### Estimation of photon-equivalent dose

Based on previously published literature on damage induced by different radiation qualities^10–12^, we assumed that the signals measured by genes, metabolites and blood cell counts respond to the cumulative biological damage burden from the sum of photon and neutron exposures (called photon-equivalent dose, *D*_*PE*_, for convenience). This relationship is represented by the following equation, where the neutron contribution is proportional to the neutron dose (*D*_*N*_) times some relative biological effectiveness (RBE) value for neutrons, *D*_*P*_ is the photon dose, and *D*_*PE*_(*RBE*) is the functional dependence of photon-equivalent dose on RBE:

1 $${D}_{PE}(RBE)={D}_{P}+RBE\times {D}_{N}$$

Since the neutron RBE values are likely to vary for different biomarkers such as genes and metabolites, we developed the following approach to estimate the photon-equivalent dose as a “weighted average”. We split the data randomly into training and testing halves (45 and 44 samples, respectively). On the training data, we calculated the Pearson correlation coefficient of each gene, metabolite and blood count with the photon-equivalent dose. The RBE value was adjusted so that the sum of the squares of all correlation coefficients would be maximized. In other words, the optimal RBE value was identified by maximizing the sum of the squares of all correlation coefficients with photon-equivalent dose. This approach allowed all variables with either positive or negative correlations with radiation exposure to contribute to the overall “optimal” RBE, labeled *RBE*_*opt*_. This procedure is mathematically described by the following equation, where *argmax*[] is an operation that finds the argument (RBE in this case) that gives the maximum value from a target function, *cor*() is an operation that calculates the Pearson correlation coefficient of the two arguments, *D*_*PE*_(*RBE*) is the functional dependence of photon-equivalent dose on RBE (from Eq. 1), *i* is the index that represents each investigated biomarker (genes, metabolites and blood cell counts), and *S*_*i*_ is the measured signal of each biomarker:

2 $$RB{E}_{opt}=argmax[{\sum }_{i}{cor({D}_{PE}(RBE),{S}_{i})}^{2}]$$

### ML procedure for biodosimetry analyses

We applied the *RBE*_*opt*_ value identified by the procedure described above (Eqs. 1–2) to the testing data set as well to the training data, generating the photon-equivalent dose variable in both data sets. This allowed us to set up ML-based regression or classification approaches to use the gene, metabolite and blood cell count data as predictors to reconstruct the following target variables of interest: Photon_equivalent_dose (*D*_*PE*_), Neutron_dose (*D*_*N*_), Neutron_10_percent (binary indicator for neutron dose fraction ≥ 10%, *i.e. D*_*N*_/(*D*_*N*_ + *D*_*P*_) ≥ 0.1) and Neutron_0.5_Gy (binary indicator for neutron dose ≥ 0.5 Gy, *i.e. D*_*N*_ ≥ 0.5 Gy). In this data set, Neutron_10_percent represents a binary yes/no neutrons classification, and Neutron_0.5_Gy was used as a higher potentially relevant cutoff for neutron dose.

We also performed a separate calculation where the CD19 blood cell counts was removed from the predictor list, but instead became the outcome variable to be predicted using the remaining biomarkers (genes and metabolites). This calculation was intended to be an example where the integrated biomarker panel is used to predict “biological injury” instead of a dosimetry endpoint.

The ML analyses of the training and testing data were performed in Python 3.10.5, Jupyter notebooks (https://jupyter.org/). Those genes, metabolites and blood counts that achieved ≥|0.3| Pearson correlation coefficients with Photon_equivalent_dose on training data were retained as potential predictors, and others were discarded. These retained predictors in the training and testing data sets are provided in Supplementary File 1 online (training_and_testing_tab).

To reduce multicollinearity and improve interpretability of the analysis, we also discarded those predictor variables that had strong ( ≥|0.7|) Pearson correlation coefficients with several other predictors. Sex (coded as Female = 0, Male = 1) and Time (1 or 7 days after exposure) variables were retained as potential predictors of interest.

The Boruta algorithm^13^ (in conjunction with random forest regressor or classifier models) was used to select the most important predictors. Boruta iteratively compares the importance score of each predictor with the importance score of its randomly shuffled “shadow”, in the context of a random forest model^14^. It duplicates the data set and randomly shuffles the values in each column. These shuffled values are called shadow features, and they are re-created in each iteration. The criterion for retaining features was set to performance above the 50th percentile of the randomized “shadow” features, using α = 0.05 with Bonferroni correction as a statistical threshold. Those predictor variables that did not pass this criterion were discarded.

The retained predictors that passed the Boruta screening were used to reconstruct Photon_equivalent_dose, Neutron_dose or CD19 cell counts (as regression tasks), or Neutron_10_percent or Neutron_0.5_Gy (as classification tasks). Several candidate ML algorithms (linear regression, random forest (RF)^14^, XGBoost^15^, LightGBM^16^, CatBoost^17^, elastic net^18^ and support vector machines^19^ for regression tasks, and logistic regression, CatBoost, XGBoost, random forest, K-nearest neighbors^20^ and naïve Bayes^21^ for classification tasks) were fitted to the training data with repeated cross-validation (fivefold, 10 repeats) and evaluated on testing data. For regression tasks, root mean squared error (RMSE) was used as the main metric to assess model performance, and mean absolute error (MAE) and coefficient of determination (R^2^) were also calculated. For classification tasks, balanced accuracy was used to assess model performance.

The stacking approach was used to integrate the outputs of these different ML models to generate an ensemble^22^. In this methodology, several ML methods (called level0 models) are applied to the training data with repeated *k*-fold cross validation. Predictions of each level0 model on out of sample data instances (those withheld during cross validation) are recorded. These predictions serve as inputs to train a meta-model (level1) which learns how to best combine the predictions of the level0 models to predict the outcome variable. Then the whole ensemble (level0 and level1) makes predictions on testing data.

## Results

The “weighted average” neutron RBE value over all tested biomarkers (*RBE*_*opt*_), identified by the procedure described above (Eqs. 1–2), was 1.29. This relatively small value is consistent with available literature on neutron RBE values for cell killing, organismal death, and blood cell count data^23–25^. It suggests that neutrons were somewhat, but not dramatically, more effective in modulating the gene, metabolite and blood cell count data which were measured in this study. We applied this estimated RBE value to the testing data set as well, generating the photon-equivalent dose.

**Figure 1:**
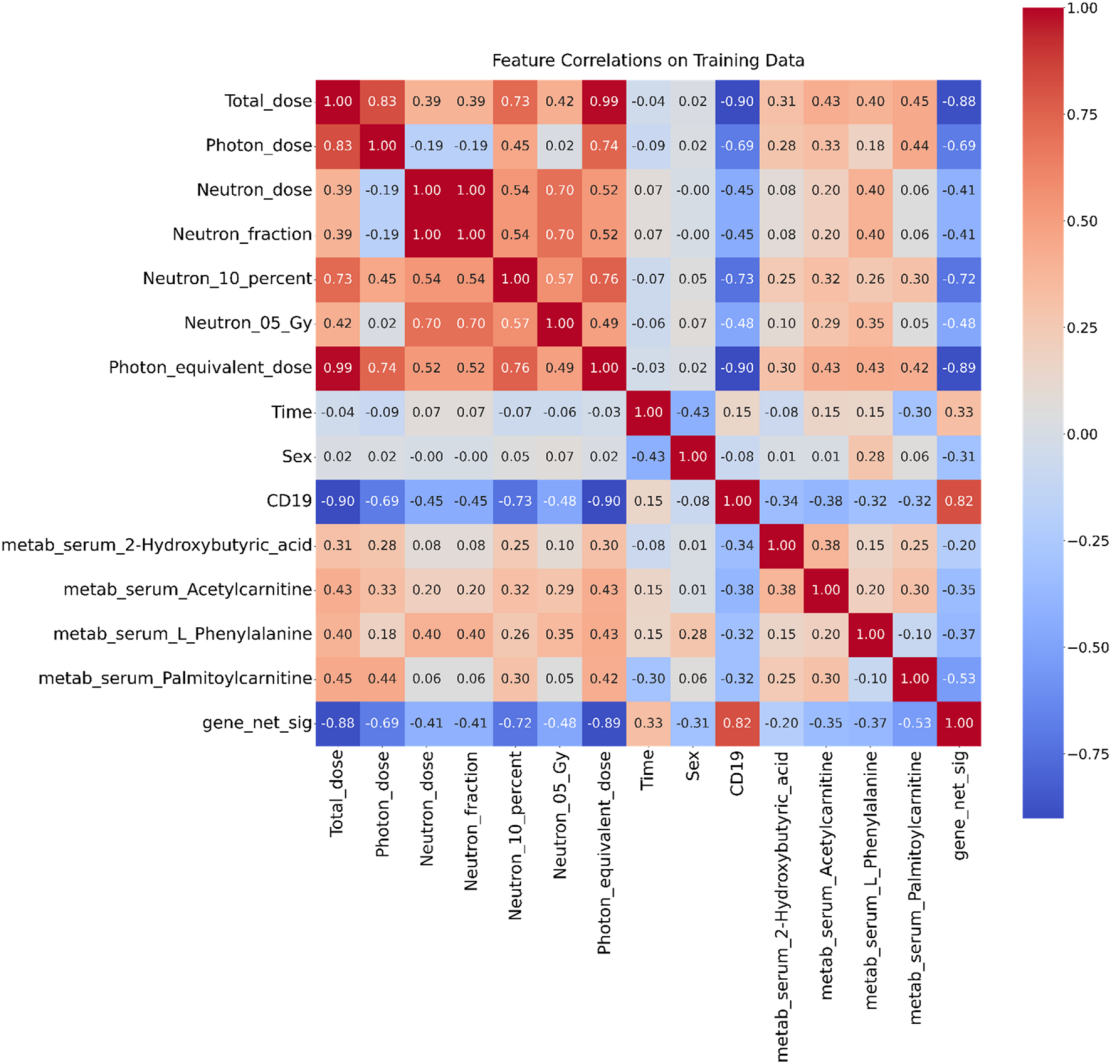
Pearson correlation matrix of all variables (features) in the training data set. Correlation coefficients are indicated by numbers, and also by the color scale.

To examine how the main variables of interest are related to each other, we constructed a matrix of Pearson correlation coefficients on the training data (Fig. 1). These results suggest that blood cell counts (CD19) and gene net signal (gene_net_sig) are strongly correlated with photon-equivalent dose and with most of the other dosimetric variables. Several metabolites are moderately correlated with these variables as well. In contrast, sex and time tended to have low correlations with the dosimetric variables.

The results of feature selection using the Boruta algorithm on training data, for the various evaluated endpoints, are summarized in Table 1. These findings are generally consistent with the correlation matrix (Fig. 1): gene_net_sig and CD19 tended to be selected as strong predictors, whereas time and sex were not. Importantly, some metabolites (*e.g.* serum phenylalanine) were also retained as strong predictors in many cases, which indicates that biomarkers from all the tested categories (genes, metabolites and blood cell counts) contributed to improving the performance of biodosimetric ML models.

**Table 1:** Summary of feature selection results for different biomarkers and endpoints, based on the Boruta algorithm. + indicates that the biomarker performed significantly better than noise variables and was retained by Boruta, whereas – indicates that the biomarker performed in the noise range and was discarded.

Biomarker	Biomarker category	Endpoints			
		Neutron 10%	Neutron 0.5 Gy	Photon-equivalent dose	CD19 cells
CD19	Blood cell counts	+	+	+	NA
Serum 2-hydroxybutyric acid	Metabolites	−	−	−	+
Serum acetylcarnitine	Metabolites	+	−	−	+
Serum phenylalanine	Metabolites	+	+	+	−
Serum palmitoylcarnitine	Metabolites	+	−	−	+
Net_sig	Genes	+	+	+	+
Time	Demographics	−	−	−	−
Sex	Demographics	−	−	−	−

**Figure 2:**
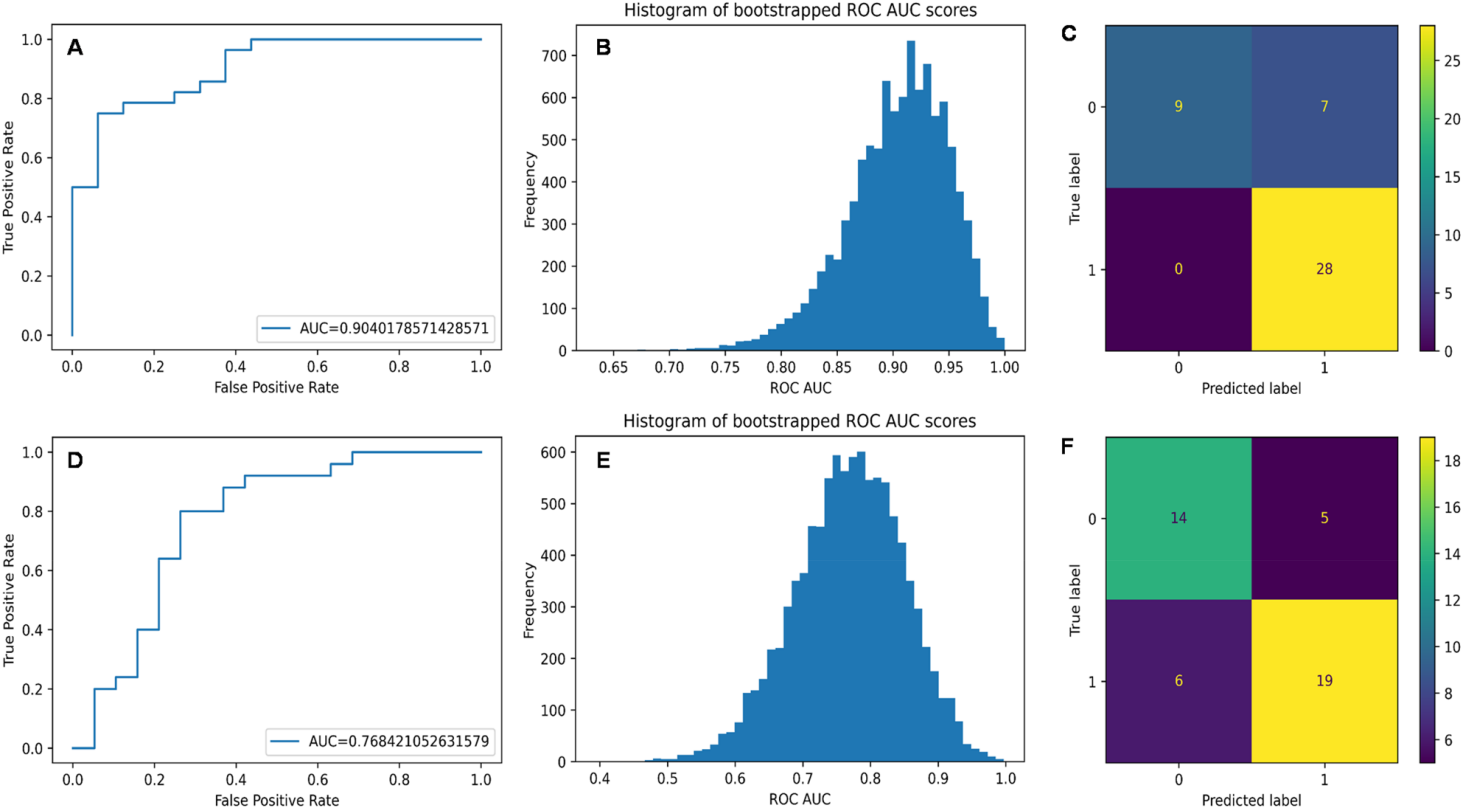
Performance of the stacking ensemble for classifying samples as exposed to ≥ 10% neutrons *vs*. < 10% neutrons (panels **A**–**C**), or ≥ 0.5 Gy neutrons *vs*. < 0.5 Gy neutrons (panels **D**–**F**). These performance metrics were calculated on testing data. Panels **A** and **D** show the ROC curves, panels **B** and **E** show histograms of ROC curve AUC values generated over 10,000 bootstrap replicates, and panels **C** and **F** show confusion matrices for model predictions *vs*. true labels.

The performance of stacking ensembles to classify samples based on neutron contribution to the exposure on testing data is shown in Fig. 2. Good classification was achieved for detecting neutron contributions ≥ 10%, which correspond to ≥ 0.3 Gy based on our experimental conditions (Fig. 2 A-C): receiver operating characteristic (ROC) area under the curve (AUC) on testing data was 0.904 (95% CI: 0.821—0.969), and classification accuracy was 84.1%. A detailed analysis of classification errors for the Neutron_10_percent on the testing data set is provided in Supplementary_File_2 online. It shows that, due to the composition of the data set, model performance metrics are somewhat inflated by the fact that it is easy for the model to distinguish between unexposed (0 Gy) and exposed (3 Gy) samples, but it is much more difficult to distinguish which of the exposed samples were irradiated with neutrons. Therefore, the majority of the classification errors (6 out of 7) on testing data were made in the subgroup of samples exposed to 3 Gy of photons, whereas all other subgroups were accurately classified always or almost always (Supplementary_File_2). The overall sensitivity of the classification was 81.8% and specificity was 90.9% on all samples. If only 3 Gy exposed samples were included (and 0 Gy controls excluded), sensitivity remained unchanged, but specificity was reduced to 75.0% (Supplementary_File_2). These results suggest that the current modeling results are promising, but contain weaknesses which could be addressed by future studies with a larger variety of neutron + photon dose combinations (*e.g.* lower and higher than 3 Gy).

Somewhat weaker, but sufficient, performance was achieved for detecting neutron contributions at a higher cutoff of ≥ 0.5 Gy (Fig. 2 D-F): ROC curve AUC on testing data was 0.768 (95% CI: 0.627—0.894), classification accuracy was 75.0%. Raising the neutron detection threshold to very high values such as ≥ 80% produced poor results (ROC curve AUC of only 0.603, Supplementary Fig. 1).

**Figure 3:**
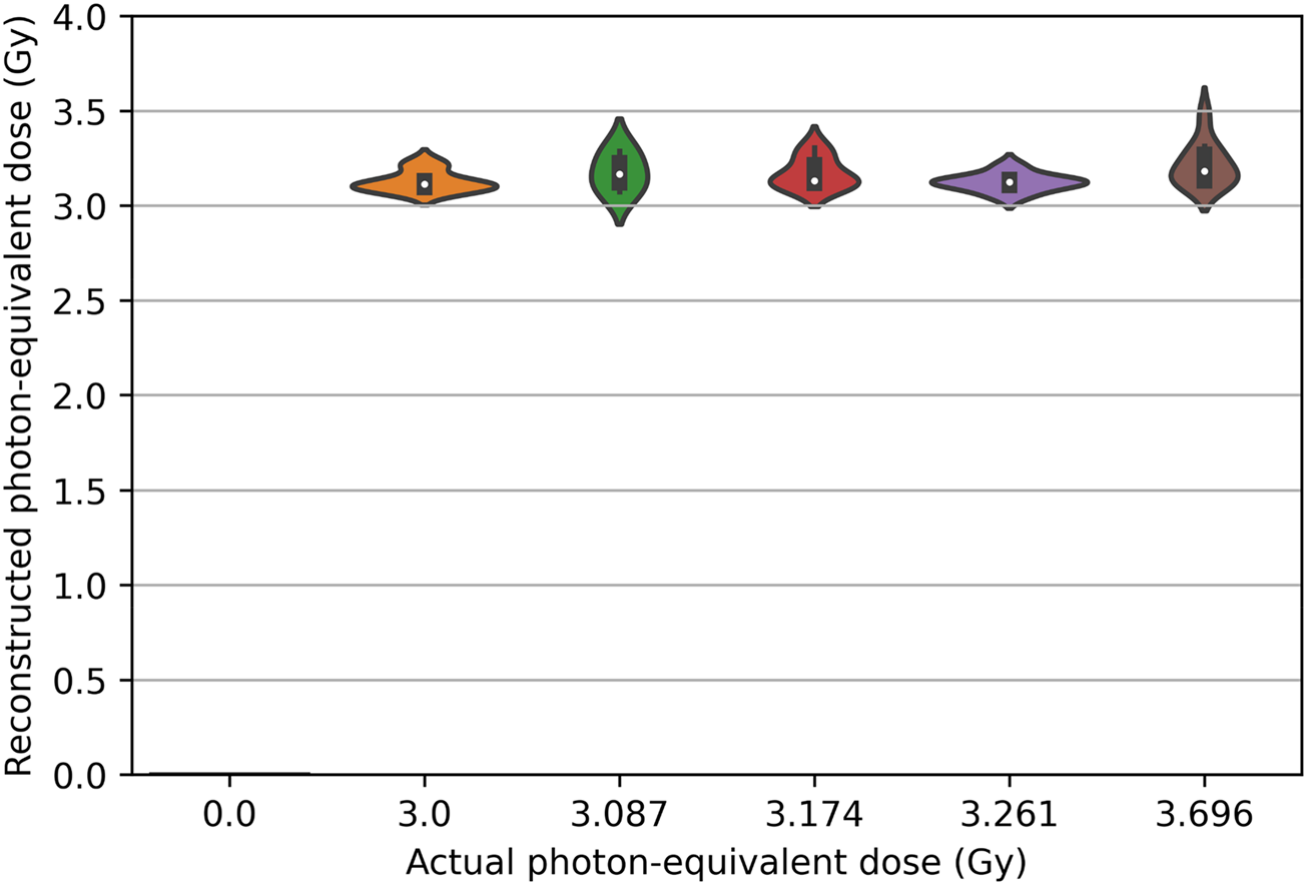
Visualization of actual and reconstructed photon-equivalent dose distributions on testing data using a violin plot.

The ability of stacking ensembles to perform a regression task to predict the photon-equivalent dose is shown in Fig. 3. The performance metrics on testing data were strong: R^2^ = 0.964, RMSE = 0.265 Gy and MAE = 0.187 Gy. However, these numbers are likely to be exaggerated due to experimental design details, and to the relatively low estimated RBE value for neutrons discussed above. The 5 exposed groups each received a total dose of 3 Gy composed of different neutron + photon mixtures, but because the neutron RBE estimate was low, photon-equivalent doses for these groups were not very different, ranging between 3.00 and 3.70 Gy (Fig. 3). The exposed groups were easily distinguished from the unirradiated control group by the ML ensemble, but the distributions of photon-equivalent dose predictions for exposed groups were clearly overlapping with each other (Fig. 3). Consequently, an attempt to reconstruct the numerical neutron dose contribution was not successful (Supplementary Fig. 2). We believe that future studies with other total dose values in addition to 3 Gy would likely be needed to develop ML models that can reliably quantify the neutron contribution using the biomarker panel developed here.

**Figure 4:**
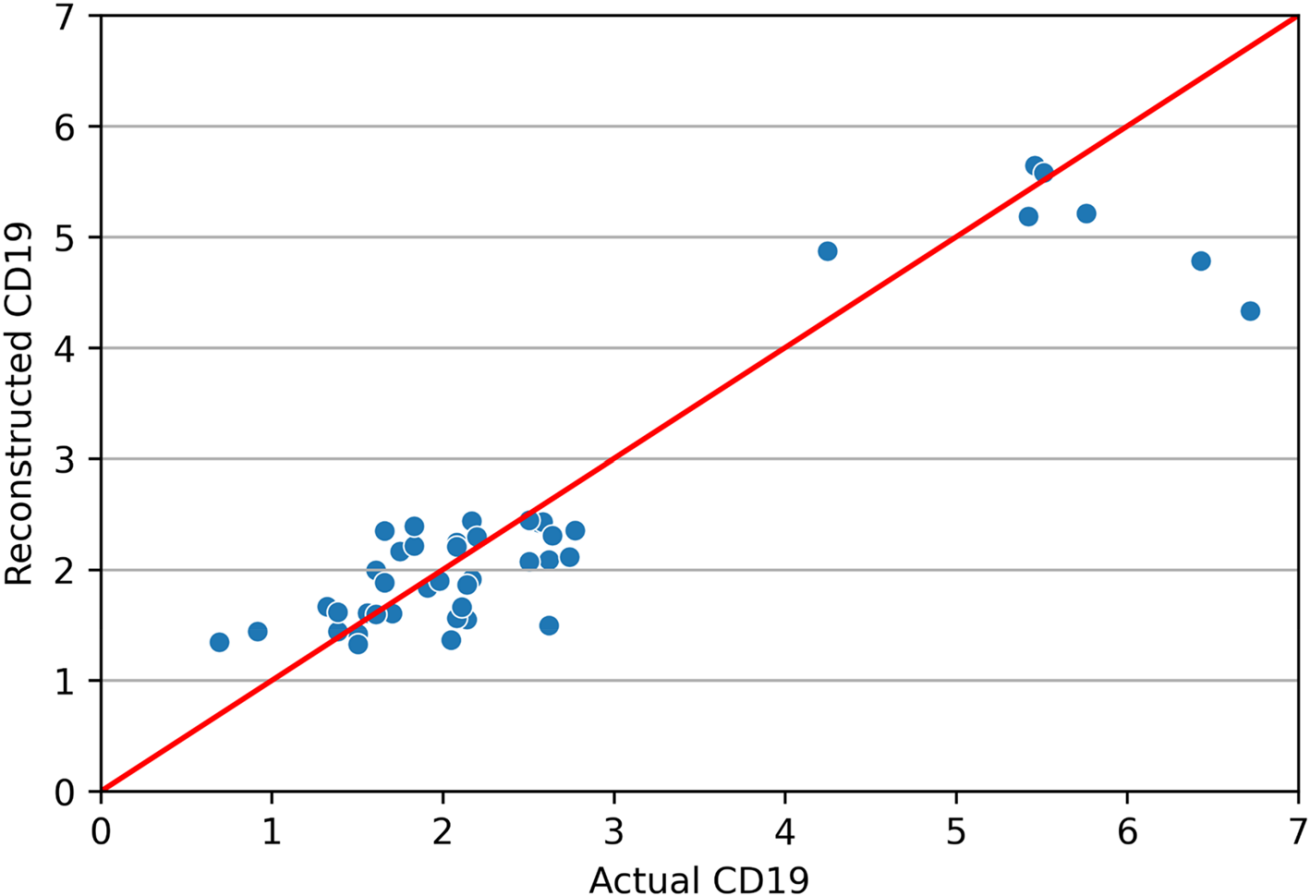
Visualization of actual and reconstructed ln-transformed CD19 blood cell count values on testing data.

The ML ensemble approach in regression mode was also able to predict blood cell counts (ln-transformed CD19 cell counts) relatively well: R^2^ = 0.849, RMSE = 0.593 and MAE = 0.409 on testing data. The comparison of actual and reconstructed values on testing data is shown in Fig. 4. This result is not surprising, since some of the variables used as predictors (*e.g.* gene_net_sig) were strongly correlated with CD19 cells (Fig. 1).

Performance of the stacking ensemble when blood cell counts (CD19) were excluded from the set of predictor variables is shown in Supplementary Fig. 3. For classifying samples as exposed to ≥ 10% neutrons vs. < 10% neutrons, calculated on testing data, the ROC AUC without CD19 was 0.810 (95% CI: 0.678, 0.923). Comparisons of actual and reconstructed photon-equivalent dose distributions on testing data produced the following performance metrics were: R^2^ = 0.961, RMSE = 0.254 Gy, MAE = 0.182 Gy. Therefore, the classification performance was reduced when blood cell counts were excluded, but not dramatically. The dose reconstruction performance remained almost the same.

## Discussion

We hypothesized that combining several radiation biomarker classes (genomics, metabolomics, CBCs) using ML will improve the accuracy of our radiation biodosimetry assays. The rationale for this concept is based on several potential strengths of combining multiple radiation biomarkers, such as the following. Combining multiple biomarkers can increase the overall sensitivity of the biodosimetry system, which can increase the ability to detect radiation exposure at lower doses or earlier time points. Combining biomarkers can also increase the specificity of the assay by reducing the likelihood of false positive results that can arise from the use of a single biomarker. Importantly, different biomarkers can provide complementary information about the biological effects of radiation exposure. For example, some biomarkers may provide information about DNA damage, while others may provide information about inflammation or cell death. Combining several biomarkers can also improve the ability to predict health outcomes following radiation exposure. By measuring multiple biomarkers, it may be possible to identify individuals who are at increased risk of developing radiation-related illnesses, such as cancer. Using multiple biomarkers can potentially reduce the effects of inter-individual variability in radiation responses, where “outlier” responses for some biomarkers (which differ substantially from the general population) can be “compensated” by more typical responses for other biomarkers in the same individual because it is unlikely that a given individual will have aberrant responses to multiple different biomarkers. Overall, combining multiple radiation biomarker assays can provide a more comprehensive and accurate assessment of radiation exposure and its biological effects, which can be useful for monitoring radiation exposure and predicting health outcomes in exposed populations.

Here, we utilized this approach to examine a complex neutron + photon exposure in mice. The rationale for this hypothesis was based on the idea that different biomarkers can provide partially complementary (rather than redundant) information, which can be harnessed by ML algorithms. We achieved our objectives by: (1) detecting neutron contributions ≥ 10%, (2) quantifying photon-equivalent dose, (3) quantifying radiation-induced changes in CD19 cell counts. Overall, this study provides a promising avenue further exploration of biodosimetric techniques based on biomarker integration using ML approaches. This outcome is in line with successful uses of ML methods in a variety of medical applications^26–28^.

Stacking ML integrates the outputs of different model types and generates a robust ensemble^22^. It is designed to improve modeling performance. Achieving an improvement in performance depends on the complexity of the problem, such as whether it is sufficiently well represented by the training data and complex enough that there is more to learn by combining predictions. It is also dependent on the choice of base models and whether they are sufficiently accurate on the analyzed data, and uncorrelated in their predictions (errors). In our analysis, stacking indeed provided improvements over individual ML models, and the best results were found using RF as the level1 model for regression tasks and CatBoost as the level1 model for classification tasks.

The main strengths of the current study include: (1) a detailed experimental design, where multiple radiation-responsive biomarkers measured in serum and urine were observed following exposures to several different neutron + photon mixtures; and (2) state of the art ML implementation to integrate these biomarkers to obtain biodosimetry outputs for several outcomes, including dosimetric variables like photon-equivalent dose and surrogates for biological injury like CD19 cell counts. These are important points because the creation of biodosimetry assays that could quickly evaluate hundreds or thousands of people is required due to increased dangers of nuclear terrorism utilizing improvised nuclear devices (IND), military conflicts or reactor accidents. Mixed fields of neutrons and photons, with shielding from buildings, and proximity to the epicenter are important factors that can make radiation exposures from such sources complicated for biodosimetry assays, and our efforts at biomarker integration seek to alleviate this problem.

There are some limitations to this approach. For example, due to resource constraints, only one non-zero cumulative dose level of 3 Gy was used for the neutron + photon mixtures. Due to limitations on the amount of blood that could be taken from one mouse, blood cell counts could not be measured on the same mice as the transcriptomic and metabolomic biomarkers. An important aspect of the ML modeling results is that the relatively easy task of distinguishing unirradiated from 3 Gy irradiated samples was partially masking the more difficult task of detecting the neutron contributions of the exposures. For these reasons, most of the mistakes for detecting ≥ 10% neutrons occurred for misclassifying 3 Gy exposed photon-only samples (Supplementary_File_2). In the future, we plan to address these aspects of the study by testing additional cumulative doses and neutron + photon combinations, and measure bone marrow stem cell and progenitor cell counts as a reliable biological injury surrogate.

Another potential limitation is “redundancy” of the information provided by some of the biomarkers. For example, CD19 cells were measured as blood cell counts, but they were also measured by the mRNA gene product, as part of the transcriptomics biomarkers. However, such redundancy of information may be advantageous in some real world biodosimetry situations, where relying on only a small number of biomarkers could be more risky than using a wider panel consisting of multiple endpoint types, which can potentially provide a more robust output.

The relatively low ratio of features to samples is another limitation of the study. There were 17 serum and 12 urine metabolites that were also considered as biodosimetry biomarkers. Sex and Time variables were also included, so the total number of potential features was 7 genes + 17 + 12 metabolites + 2 demographics = 38. The total number of samples was 89, so there were 2.3 samples/feature. This ratio is actually a conservative under-estimate, because the 7 genes were not treated as independent features, but combined into one feature gene net signal. While a ratio of 2.3 samples/feature is not ideal, the impact of the sample-to-feature ratio depends on the complexity of the problem, the quality of the data, and the inherent relationships among the features. ML algorithms like random forest can handle high-dimensional data, where the number of features is even larger than the number of samples. By randomly selecting a subset of features for each tree, random forest can effectively explore the feature space and identify relevant patterns. Random forest algorithms demonstrate resilience to noisy or irrelevant features, as well as correlated features. Since each decision tree in the forest is trained independently, the impact of noise and correlations in specific features on the overall performance is reduced. The averaging or majority voting process employed by random forest further diminishes the influence of both noisy and correlated features, enhancing the algorithm's ability to capture meaningful patterns and relationships in the data.

To strengthen the analysis pipeline further, we used stacking, which involves combining the predictions of multiple machine learning algorithms, including random forest, to improve overall performance. By stacking several ML algorithms, each with its unique strengths and weaknesses, it becomes possible to exploit the diversity of their predictions and potentially achieve better predictive accuracy. The outputs of individual algorithms can be used as input features for a higher-level model, often referred to as a meta-learner or blender, which learns to make final predictions based on the combined information from the base models. Stacking allows for the utilization of different algorithms' capabilities to handle specific aspects of the data, including cases where there are more features than samples. The ensemble of models can collectively handle noise, irrelevant features, and correlations more effectively, leveraging the strengths of each component algorithm while mitigating their individual limitations.

Overall, any radiation biodosimetry methodology, particularly when measuring metabolites, genes, and blood cell counts, faces several challenges, include the following issues: The effects of radiation on metabolites, genes, and blood cell counts can vary based on factors such as radiation type, duration of exposure, and individual variability. Understanding these intricate interactions and accurately measuring their changes poses a challenge. People may exhibit variations in their responses to radiation exposure due to individual factors, including genetic predisposition, underlying health conditions, and lifestyle choices. These inter-individual differences make it challenging to develop universal biodosimetry methods that accurately reflect individual radiation doses. Differentiating radiation-induced changes from baseline variability can be difficult. Moreover, responses of metabolites, genes, and blood cell counts to radiation exposure can be dynamic over time. It is essential to capture these time-dependent changes accurately to assess radiation dose and estimate potential health risks accurately. High-dose-rate and low-dose-rate exposures may trigger different responses, which need to be considered when interpreting the biodosimetry results. Finally, translating complex biodosimetry methodologies into practical applications for real-time or retrospective dose estimation can be challenging. Implementing these methods in emergency response situations or large-scale radiation incidents requires well-established protocols, trained personnel, and efficient laboratory infrastructure. Addressing these challenges requires ongoing research and technological advancements.

In summary, here we developed a practical ML-based approach to integrate information from several radiation-responsive biomarker types and implemented it on data from mice exposed to several neutron + photon mixtures. Representatives of each tested biomarker category (transcripts, metabolites and blood cell counts) were found to contribute to improving the ML-model’s performance. Importantly, output from such biomarker assays can be obtained more quickly following radiation exposure, compared with traditional cytogenetic markers such as dicentric chromosomes. These concepts should be refined by future studies, but we believe that they are potentially applicable and promising for performing biodosimetry on human populations exposed to radiation mass casualty events.

## Supplementary Information

Supplementary Information 1.

Supplementary Information 2.

Supplementary Information 3.

## Data Availability

All datasets analyzed during the current study are available in Supplementary_File_1 online.
