# Supplementary material for: Biomarker integration for improved biodosimetry of mixed neutron + photon exposures
Source: Sci Rep. 2023 Jul 6;13:10936. doi: 10.1038/s41598-023-37906-3 (PMC10325958; doi:10.1038/s41598-023-37906-3)
Supplement: Supplementary file 1 — Supplementary Information 1. [file 41598_2023_37906_MOESM1_ESM.docx]

**Supplementary Figure 1.** **Performance of the stacking ensemble for classifying samples as exposed to ≥80% neutrons *vs*. <80% neutrons, calculated on testing data**. The left panel shows the ROC curve, and the right panel show a histogram of ROC curve AUC values generated over 10,000 bootstrap replicates.

**Supplementary Figure 2. Visualization of actual and reconstructed neutron dose distributions on testing data using a violin plot**.

**Supplementary Figure 3.** **Performance of the stacking ensemble on testing data, when blood cell counts (CD19) were excluded from the set of predictor variables.** Panel A: ROC curve for classifying samples as exposed to ≥10% neutrons *vs*. <10% neutrons, calculated on testing data. The AUC was 0.810 (95% CI: 0.678, 0.923). Panel B: Visualization of actual and reconstructed photon-equivalent dose distributions on testing data using a violin plot. The performance metrics were: R^2^ = 0.961, RMSE = 0.254 Gy, MAE = 0.182 Gy.
